# Increased osteoclastogenesis contributes to bone loss in the Costello syndrome *Hras G12V* mouse model

**DOI:** 10.3389/fcell.2022.1000575

**Published:** 2022-10-18

**Authors:** Sayantan Nandi, Saravanakkumar Chennappan, Yannik Andrasch, Miray Fidan, Melanie Engler, Mubashir Ahmad, Jan P. Tuckermann, Martin Zenker, Ion Cristian Cirstea

**Affiliations:** ^1^ Institute of Comparative Molecular Endocrinology, Ulm University, Ulm, Germany; ^2^ Masonic Medical Research Institute, Utica, NY, United States; ^3^ Institute of Human Genetics, University Hospital Magdeburg, Magdeburg, Germany

**Keywords:** RASopathy, HRAS, Costello syndrome, osteoporosis, osteoclast, osteoclastogenesis

## Abstract

RAS GTPases are ubiquitous GDP/GTP-binding proteins that function as molecular switches in cellular signalling and control numerous signalling pathways and biological processes. Pathogenic mutations in *RAS* genes severely affect cellular homeostasis, leading to cancer when occurring in somatic cells and developmental disorders when the germline is affected. These disorders are generally termed as RASopathies and among them Costello syndrome (CS) is a distinctive entity that is caused by specific *HRAS* germline mutations. The majority of these mutations affect residues 12 and 13, the same sites as somatic oncogenic *HRAS* mutations. The hallmarks of the disease include congenital cardiac anomalies, impaired thriving and growth, neurocognitive impairments, distinctive craniofacial anomalies, and susceptibility to cancer. Adult patients often present signs of premature aging including reduced bone mineral density and osteoporosis. Using a CS mouse model harbouring a *Hras G12V* germline mutation, we aimed at determining whether this model recapitulates the patients’ bone phenotype and which bone cells are driving the phenotype when mutated. Our data revealed that *Hras G12V* mutation induces bone loss in mice at certain ages. In addition, we identified that bone loss correlated with an increased number of osteoclasts *in vivo* and *Hras G12V* mutations increased osteoclastogenesis *in vitro*. Last, but not least, mutant osteoclast differentiation was reduced by treatment *in vitro* with MEK and PI3K inhibitors, respectively. These results indicate that Hras is a novel regulator of bone homeostasis and an increased osteoclastogenesis due to *Hras G12V* mutation contributes to bone loss in the Costello syndrome.

## 1 Introduction

RAS (rat sarcoma) proteins are small GDP/GTP binding proteins that act as molecular switches of many signalling pathways. In their active state, they control numerous signalling pathways to regulate a wide variety of biological processes including proliferation, differentiation, and apoptosis. Due to their importance in cancer, RAS GTPases along with their downstream pathways such as the mitogen-activated protein kinase (MAPK) and phosphatidylinositol-3-kinase (PI3K)-protein kinase B (PKB/AKT) pathways have been extensively studied. RAS somatic mutations at hot spot codons 12, 13, and 61 are associated with more than 30% of human cancers (Karnoub and Weinberg, 2008), while germline RAS mutations cause a group of developmental disorders generally termed RASopathies. *KRAS* (Kirsten RAS) germline mutations are associated with Noonan syndrome (NS; MIM#163950) and cardio-facio-cutaneous syndrome (CFCS; MIM#115150), *NRAS* (neuroblastoma RAS), *RRAS* (RAS-related), *MRAS* (muscle RAS) and *RIT1* (RAS-like protein expressed in many tissues 1) also lead to Noonan syndrome, whereas *HRAS* germline mutations cause Costello syndrome (CS; MIM#218040) (Aoki et al., 2005; Kratz et al., 2006; Schubbert et al., 2006; Cirstea et al., 2010; Flex et al., 2014; Higgins et al., 2017; Niihori et al., 2019). *HRAS* (Harvey RAS) mutations identified in CS predominantly affect the same residues that are frequently mutated in cancer. Mutations affecting amino acids 12 and 13 were identified in approximately 90% of the patients, with glycine 12 substitution to serine (G12S) being the most frequent CS-associated mutation (Aoki et al., 2005; Kerr et al., 2006; Gripp et al., 2011). In contrast to G12S, less frequent G12V, G12A, and G12D mutations are associated with a more severe expression of the phenotype lead to early lethality (Burkitt-Wright et al., 2012; Lorenz et al., 2012; Giannoulatou et al., 2013). CS is characterized by cardiac anomalies (pulmonary stenosis, septal defects, hypertrophic cardiomyopathy), short stature, failure to thrive, distinctive craniofacial anomalies, loose skin, musculoskeletal abnormalities, relative macrocephaly, mild to severe intellectual disability, and predisposition to tumours (Aoki et al., 2005; Kratz et al., 2011; Kratz et al., 2015; Tidyman, Lee and Rauen, 2011). The adult phenotype of CS has striking features of premature aging illustrated by an aged facial and skin appearance, hair loss, osteopenia, osteoporosis, postural deficit, reduced muscle strength, and increased incidence of benign and malignant tumours (White et al., 2005). CS patients, as well as other RASopathy patients develop osteoporosis at a relatively young age (White et al., 2005; Choudhry et al., 2012), suggesting that proper RAS regulation is important for maintaining bone mass.

Osteoporosis is a major public health issue in modern society with an increased elderly population. It is characterized by a low bone mass that increases the fracture risk (Svedbom et al., 2013). A tightly balanced cross-talk between osteoblasts, osteocytes and osteoclasts is critical for proper bone remodelling to preserve bone mass (Raggatt and Partridge, 2010; Teitelbaum, 2011; Long, 2012; Teti, 2013; Xu and Teitelbaum, 2013; Katsimbri, 2017). In CS and other RASopathies, osteoporosis was demonstrated by dual-energy x-ray absorptiometry (DEXA) and increased amounts of matrix degradation products (e.g., deoxypyridinoline, DPD) in the urine (White et al., 2005; D. Stevenson et al., 2011; Stevenson and Yang, 2011). The increased amount of bone resorption products suggests either an increased osteoclastogenesis and/or increased osteoclastic activity, but so far there is no data clearly supporting abnormalities of osteoclasts on a cellular level in CS. Osteoclast dysregulation in RASopathies is indirectly suggested by studies of Neurofibromatosis Type 1 (NF1), where haploinsufficiency of RAS negative regulator *NF1* gene leads to an increased RAS activation. Increased RAS signalling due to *NF1* loss in a mouse model was found to be associated with an increased number of osteoclast progenitor cells and multinucleated osteoclasts, as well as increased lytic activity (Yang et al., 2006). Studies with *Nf1*-deficient mice further highlighted a role for RAS GTPases in osteoblast homeostasis, where mouse mesenchymal progenitor cells differentiation into osteoblast is defective, and this may contribute to bone abnormalities observed in NF1 patients (Wu et al., 2006). Deletion of *Nf1* in mouse osteoblast progenitor cells led to reduced bone mineralization and an increased number of osteoclasts, indicating that an enhanced activation of RAS-MAPK in osteoblasts could support and increased osteoclastogenesis (Elefteriou et al., 2006). Recently, involvement of osteoblasts in CS bone homeostasis was suggested by a study that evaluated *in vitro* osteoblast differentiation potential of CS patient-derived induced pluripotent stem cells (iPSC). In this study, increased RAS signalling due to HRAS G12S mutation impaired osteoblast differentiation and osteogenesis without affecting mesenchymal stem cells. (Choi et al., 2021). In contrast to HRAS, KRAS hyperactivation increases bone mass by increasing mouse osteoblast progenitor cell proliferation *via* MAPK and AKT pathways without affecting mature osteoblasts (Ge et al., 2009; Li et al., 2017).

To date, the underlying causes of osteoporosis in CS patients and CS mouse models are largely unknown. Furthermore, there are some contradicting reports between clinical data suggesting an increased bone resorption (Stevenson et al., 2011) and *in vitro* studies using CS iPSCs-differentiated osteoblasts that revealed that osteogenesis is affected (Choi et al., 2021). Therefore, in this study, utilizing the CS mouse model harbouring an *Hras G12V* germline mutation (Schuhmacher et al., 2008) we aimed at proving that these mice recapitulate the patients’ phenotype and undergo bone loss, as well as to identify which bone cell type is driving the bone phenotype when Hras is mutated. Long term analyses revealed that CS mice undergo bone loss and this is triggered by an increased osteoclastogenesis. Unexpectedly, we also observed that bone mass was not affected in CS mice that survive beyond median lifespan and the reason behind this observation is still elusive. Inhibitory treatments using MEK and PI3K inhibitors reduce Hras G12V-induced osteoclast differentiation *in vitro*, thus suggesting a pivotal role for both pathways. Thus, targeting these pathways could serve as a therapeutical intervention for phenotype rescue and future clinical studies.

## Material and methods

### Mouse husbandry

*Hras G12Vgeo* mice were previously described (Schuhmacher et al., 2008) and were a kind gift from Prof. Mariano Barbacid (CNIO, Spain) and they were backcrossed and maintained in the C57BL/6 background. The cohort containing the hetero- and homozygous knock-in mutation for *Hras G12V*, were monitored over a period of more than 2 years. Genomic DNA isolated either from tail or ear clips was amplified by PCR with HrasF-B1 (5′-TCT AAT TTG GGT GCG TGG TTG-3′) and HrasR-B2 (5′- CCA CTT GAG ACG GCT AAT AGA TGC-3′) primers for *Hras* allele. PCR products were separated by electrophoresis on a 2% (W/V) agarose gel in order to identify specific bands of 230 bp corresponding to the wild type (+) and 430 bp for the knock-in (KI) *Hras G12V* alleles. Two to five mice per cage were housed under specific pathogen-free conditions in an environment of 22 ± 2°C with a 12 h light:12 h dark cycle, with free access to water and nutritionally balanced diet (*ssniff*, Spezialdiäten GmbH). The animals were weighed every month and scored for pathologies and scoring frequency was performed daily when pathologies arise in mice cohorts.

### Micro-computed tomography

Femurs and vertebrae were collected from littermates CS mutant and control mice with defined age, were fixed in 4% paraformaldehyde (PFA) for 48 h and were placed 0.5% PFA for long term storage. Bone mass parameters were analysed with Skyscan 1174 compact X-ray micro-CT (Bruker), equipped with an X-ray source functioning of 50 KV/6.5 µm spatial and a 1.3-megapixel X-ray camera, which was used at a rotation step of 0.5°. Bone mineral density (BMD) and tissue mineral density (TMD) measurements were measured using Skyscan 1176 high resolution *in vivo* micro-CT (Bruker) equipped with an X-ray source of 90 KV/9 µm spatial resolution and a 11-megapixel X-ray camera, at 1° rotation step. For analysis of femurs, the region of interest was defined at either 0.7 mm from the distal growth plate (trabecular bone) or 3.3 mm (cortical bone). For trabecular bone, bone volume/tissue volume (BV/TV %), trabecular thickness (Tb. Th.), trabecular separation (Tb. Sp.), trabecular number (Tb. N.) were measured, while for cortical bone only cross-sectional thickness (Cs. Th.) was determined, according to guidelines set by the ASBMR histomorphometry nomenclature committee (Dempster et al., 2013).

### *Ex vivo* bone cells quantification

Osteoblasts, osteocytes and osteoclast quantification was performed on TRAP (tartrate-resistant acid phosphatase)-stained sections. Bones were decalcified in 15% EDTA for 10–14 days, embedded in paraffin and 5 µm sections (Leica RM2255 microtome, Leica Biosystems) were stained for TRAP as described (Madsen et al., 2011). Sections were deparaffinized at 60°C for 15 min, placed twice in xylene, rehydrated in decreasing series of ethanol (twice for each ethanol concentration of 100%, 90%, 70%, respectively) and placed in demineralized water. After a pre-incubation in TRAP buffer for 10 min and incubation in TRAP staining solution for 60 min at 37°C, sections were washed with tap water and counterstained with hematoxylin for 2 min. Finally, slides were mounted with Aquatex aqueous mounting media (Merck-Millipore). Osteoclasts appear as reddish-pink, while all the other bone cells appear as blue.

Imaging of TRAP sections was done using a Leica DMI6000B fully automated inverted microscope (Leica Biosystems) and osteoclasts, osteoblasts and osteocytes were then quantified using the OsteoMeasure software (Osteometrics, Decatur). TRAP-positive multinuclear cells (≥3 nuclei) were designated as osteoclasts (Ghayor et al., 2011). Osteoblasts and osteocytes were quantified from the same stainings and were differentiated based on their TRAP-negative staining, morphology and location in the bone. For osteoclasts, parameters measured were osteoclast number per bone perimeter (Oc. N./B.Pm.) and osteoclast surface per bone surface (Oc.S./B.S.). Osteoblasts were identified based on their cuboidal morphology and location on bone surface (Watson, 1976; Maynard and Downes, 2019) and osteoblast number per bone perimeter (Ob.N./B.Pm) and osteoblast surface per bone surface (Ob.S./B.S.) were measured. Osteocytes were also identified based on their special morphology and location inside long bones (Vatsa et al., 2008) and osteocyte number (Ot.N.) was quantified.

### Quantification of bone turnover products in mouse plasma

Blood was collected by heart puncture and was then centrifuged at 5,000 rpm for 12 min to separate the plasma, which was further used for measurements of bone turnover products. Bone degradation product C-terminal telopeptide of type I collagen (CTX-I) was measured using serum cross-laps (CTX-I) ELISA (Immunodiagnostic Systems), while bone formation product procollagen type I N-propeptide (P1NP) ELISA was measured using Rat/Mouse P1NP EIA (Immunodiagnostic Systems), both following the manufacturer’s instructions.

### Osteoclast *in vitro* differentiation and quantification

Bone marrow-derived cells were freshly isolated from femur and tibia by flushing the marrow using α-MEM supplemented with 1% Penicillin/Streptomycin and 10% FCS (Life Technologies). Cells were seeded in 24-well plates at a density of 250 000 cells/well and their proliferation was stimulated with 25 ng/ml macrophage colony-stimulating factor (M-CSF, R&D Biosystems) for the rest of the experiment. After 3 days, differentiation was stimulated by supplementing culture media with 50 ng/ml receptor activator of nuclear factor kappa-Β ligand (RANKL, R&D Biosystems). Cells were stimulated with RANKL and M-CSF every second day and after 10 days post-seeding, cells were fixed with 4% PFA and stained for TRAP using Leucocyte acid phosphatase kit (Sigma Aldrich), according to manufacturer’s protocol. Multinucleated TRAP positive cells (nuclei number ≥3) were imaged with Leica DMI 6000B microscope and their number and total area were quantified using the OsteoMeasure software (OsteoMetrics, Decatur).

To study the impact of MEK (U0126) or PI3K (LY294002) inhibition on osteoclast differentiation, U0126 (Selleckchem) were added to a final concentration of 5 µM and LY294002 2.5 µM, respectively, at various time points of differentiation and its effects on osteoclast differentiation were monitored by quantification of osteoclast number and area from images of TRAP staining, as described above.

### Osteoclast *in vitro* resorption assays

250000 bone marrow derived monocytes were seeded in a 24-well plate Corning OsteoAssay surface plate. Osteoclast differentiation protocol was performed as described above. At the end of differentiation time, cells were washed with 1X PBS for 3 times and incubated with 5% solution of sodium hypochlorite for 10 min at RT to remove the cells. Next, plates were air dried at RT and resorption areas for each well were acquired automatically using Leica DMI6000B. Resorbed areas were quantified with ImageJ and plotted using GraphPad Prism 8.

Alternatively, osteoclasts resorptive capacities were done by a pit assay on bovine cortical bone slices (Immunodiagnostic Systems). 250000 bone marrow-derived cells/well were seeded in a 24-well plate containing the cortical bone slices. At the end of differentiation time, media was removed carefully from the wells by aspiration and bone slices were incubated with ammonium hydroxide solution overnight. Next, cells were removed from bone slices by ultrasonication for 1 min (Transsonic T460, Elma) and slices were stained with 0.5% Toluidine Blue. To visualize resorption pits, bone slices were imaged with Olympus BX41 equipped with Olympus DP72 camera at ×10 magnification. Osteoclast number and total area were quantified with the OsteoMeasure software (Osteometrics, Decatur) and plotted using GraphPad Prism 8.

### Statistical analyses

Data are presented as mean ± standard deviation (SD) or mean ± standard error of the mean (SEM), unless specified, with number of animals or cells used functional experiments presented as *n* = x. Group sizes were determined based on sample availability and no statistical method was used to predetermine the sample size. All statistical analyses were performed using Prism V7, V7.0 or V9.0 (GraphPad Software). Differences between two groups were analysed using the one-tailed or two-tailed Student’s t-test. Data were considered to be significantly different if *p* < 0.05 and were mentioned in either appropriate text or in legends.

## Results

### *Hras G12V* mutation induces bone loss

**FIGURE 1:**
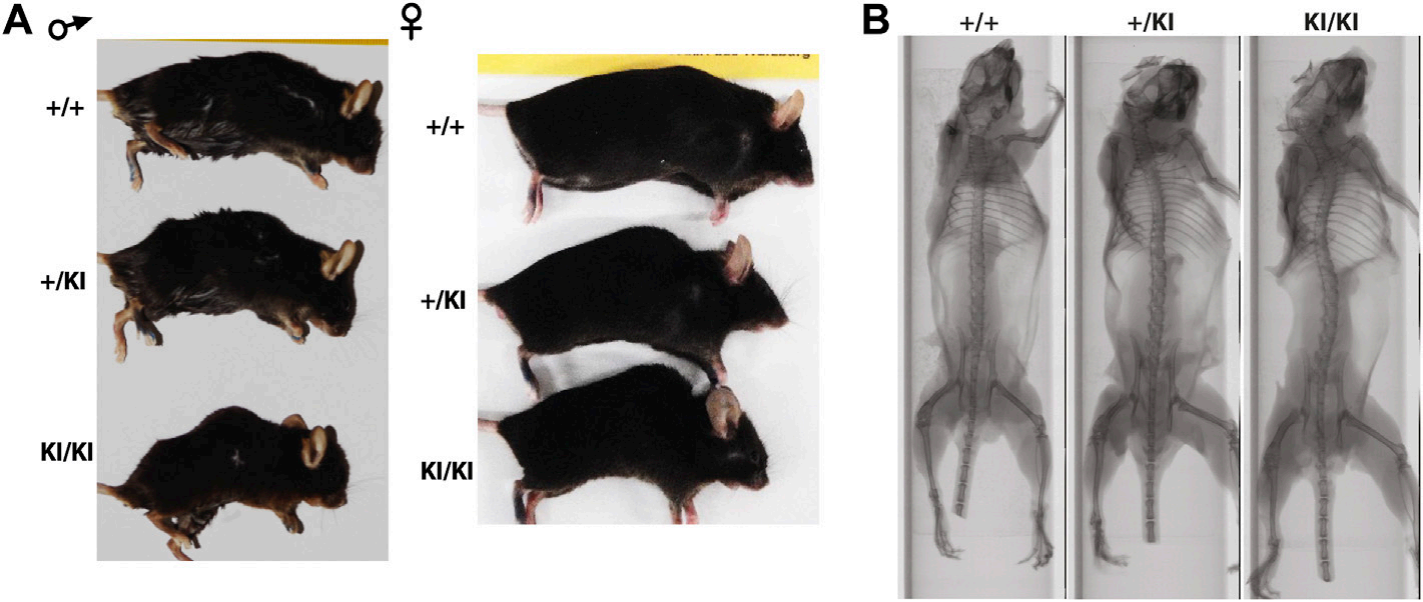
CS *Hras G12V* mouse model representative images of the external morphology **(A)** and micro-computed tomography of the vertebral column and long bones in 15-month-old females **(B)**.

Clinical studies in the CS and other RASopathies have suggested that RAS-MAPK gain-of-function mutations are associated with osteopenia and osteoporosis (Susan M White et al., 2005; D. Stevenson et al., 2011; Choudhry et al., 2012). To study whether osteoporosis occurs in the CS *Hras G12V* mouse model we monitored cohorts of single knock-in (+KI) and double knock-in (KI/KI) CS mice of both sexes together with wild type (+/+) littermates control mice. We observed a prominent abnormal backbone curvature of the vertebral column, thus phenocopying skeletal abnormalities seen in patients (Figures 1A,B).

The first analysis we performed was to monitor whether changes in the tissue (TMD) and bone (BMD) mineral densities occur in our age and gender cohorts. Micro-computed tomography (µCT) did not identify any changes in BMD and TMD between CS and control mice irrespective of their age and gender by µCT (Supplementary Figure S1).

**FIGURE 2:**
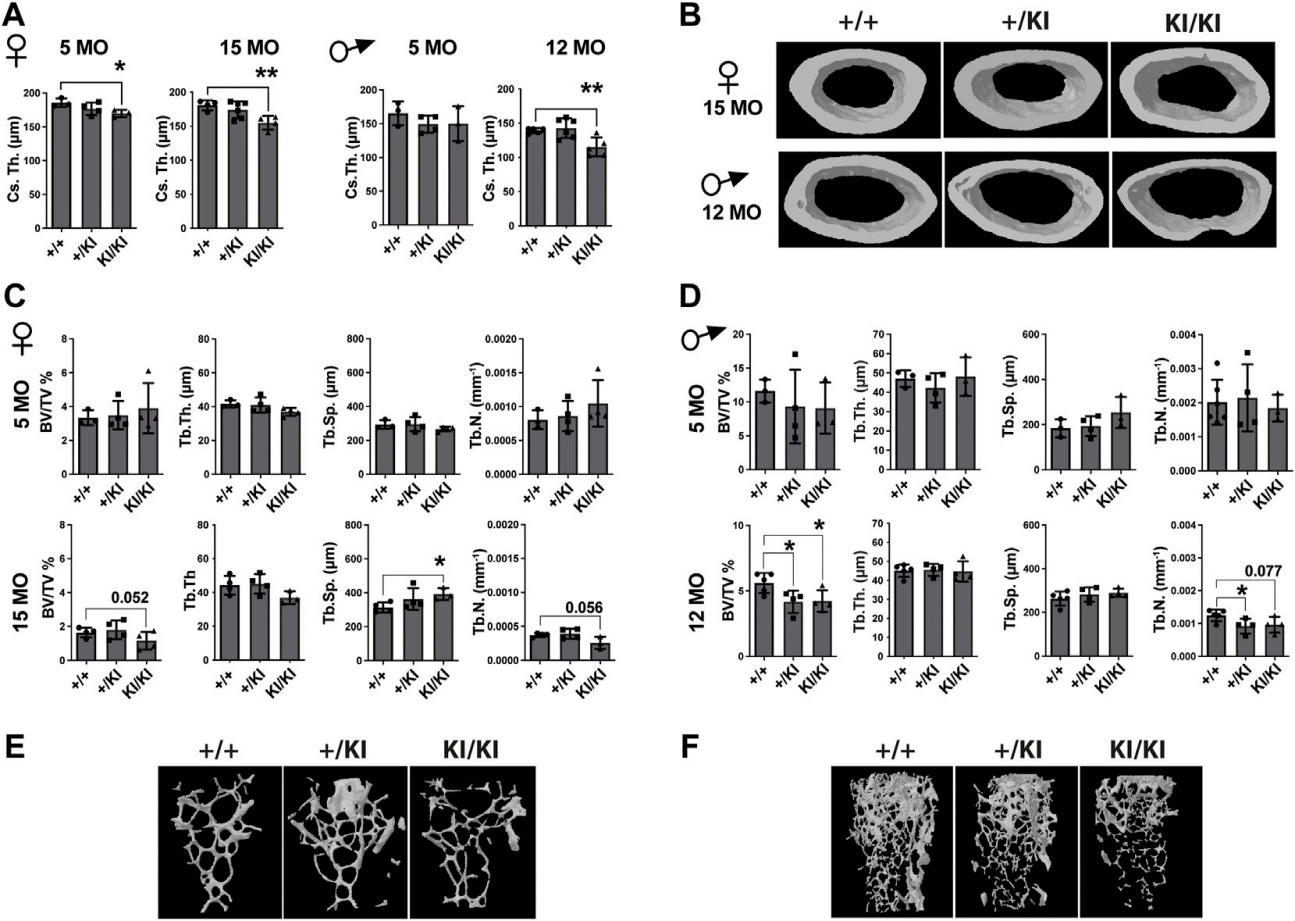
*Hras G12V* mutation trigger a femoral bone loss in CS mice at defined ages and CS mice that survive beyond median lifespan are not affected by a bone loss. **(A)** µCT measurements of cortical bone in CS females and males. **(B)** 3D reconstructed cortical bone images from 15- and 12-month-old females and males, respectively. µCT measurements of trabecular bone in CS females **(C)** and males **(D)**. 3D reconstructed cortical bone images of 15-month-old females **(E)** and 12-month-old-males **(F)**. 5 months females (n = 3–4), 15 months females (n = 4), 5 months males (n = 3–4), 12 months males (n = 34–5). Data are shown as mean ± SEM. Statistical differences were analysed by unpaired 2-tailed student’s t-test where *p < 0.05, **p < 0.01.

As CS mice appear smaller than their wild type counterparts (Figure 1A), the reduction of bone mass in CS mice may well be proportional to bone size. However, measurements of tibial bone length in both male and female mice revealed similar tibia length among mice from same age and gender groups (Supplementary Figure S1A), indicating a reduction of the bone mass would not be a consequence of a reduced bone length. Next, we performed a detailed µCT analysis of bones collected from 5- and 15-month-old females and 5- and 12-month-old males that were available for this study. Analyses of the femoral cortical bone revealed a significant reduction of cross-sectional thickness in 5- and 15-month-old KI/KI female mice, whereas in males a significant reduction was observed in 12-month-old KI/KI, but not in the 5-month-old cohort (Figures 2A,B). Next, we analysed the trabecular (cancellous) bone in the same femoral bones. Bone volume/tissue volume (BV/TV) was significantly reduced as a consequence of decreased trabecular number (Tb. N) in 15-month-old with no change in 5-month-old KI/KI females (Figures 2C,E). Similarly, the 12-month-old +/KI and male mice had a significant reduction in BV/TV of the femoral trabecular bone (Figures 2D,F). While, in 15-month-old KI/KI females although the trabecular separation (Tb. Sp.) was significantly increased, the trabecular BV/TV was only trending towards a reduction (Figures 2C,E).

**FIGURE 3:**
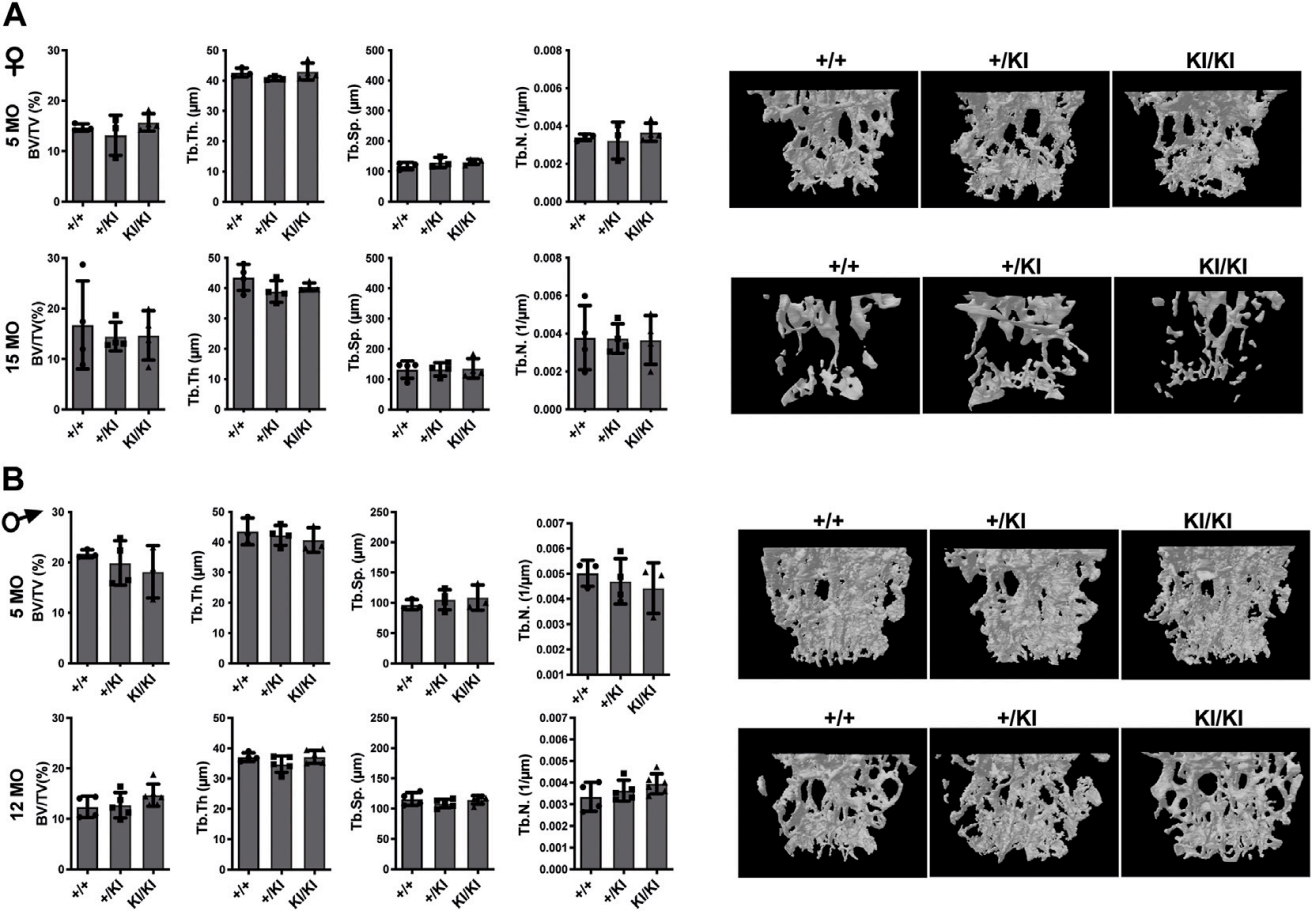
Bone loss does not occur in the L5 vertebra of CS mice **(A)** µCT measurements and three-dimensional reconstruction of L5 vertebra in CS females. **(B)** µCT measurements and three-dimensional reconstruction of L5 vertebra in CS males. Data are shown as mean ± SEM. 5-month-old-females (*n* = 3–4), 15-month-old-females (*n* = 4), 5-month-old-males (*n* = 3–4), 12-month-old-males (*n* = 4–5), 18-month-old males (*n* = 4–7). Data are shown as mean ± SEM. Statistical differences were analysed by unpaired 2-tailed student’s t-test.

In addition to femoral bone, we monitored bone mass in lumbar 5 vertebra (L5) as both mice and patients develop vertebral abnormalities. µCT measurements in L5 vertebrae did not reveal any significant differences in CS mice when compared to controls in any of the parameters tested (Figures 3A,B). A major difference between humans and mouse is that in humans the pressure exerted on L5 due to biped posture may act as an external stress factor and in combination with Hras G12V constitutive activation may have rather detrimental effects and lead to bone loss. Therefore, in addition to analysing the thickness of L5 vertebra in CS mice we measured the caudal vertebra 1 (C1) as the constant tail wiggling acts as a stress factor and may act as an enhancer of bone loss (Li et al., 2019; Scheuren et al., 2020). Our analysis showed a significant reduction of C1 vertebral BV/TV in 15-month-old KI/KI females due to a reduction of Tb. Th. (Supplementary Figure S2A). The BV/TV was trending towards a reduction in 5-month-old KI/KI females (Supplementary Figure S2A). Similarly, 12-month KI/KI males also show a reduction of BV/TV which did not reach statistical significance although the Tb. Th. was significantly decreased.

### *Hras G12V* mutation increases osteoclast number in femoral bone

**FIGURE 4:**
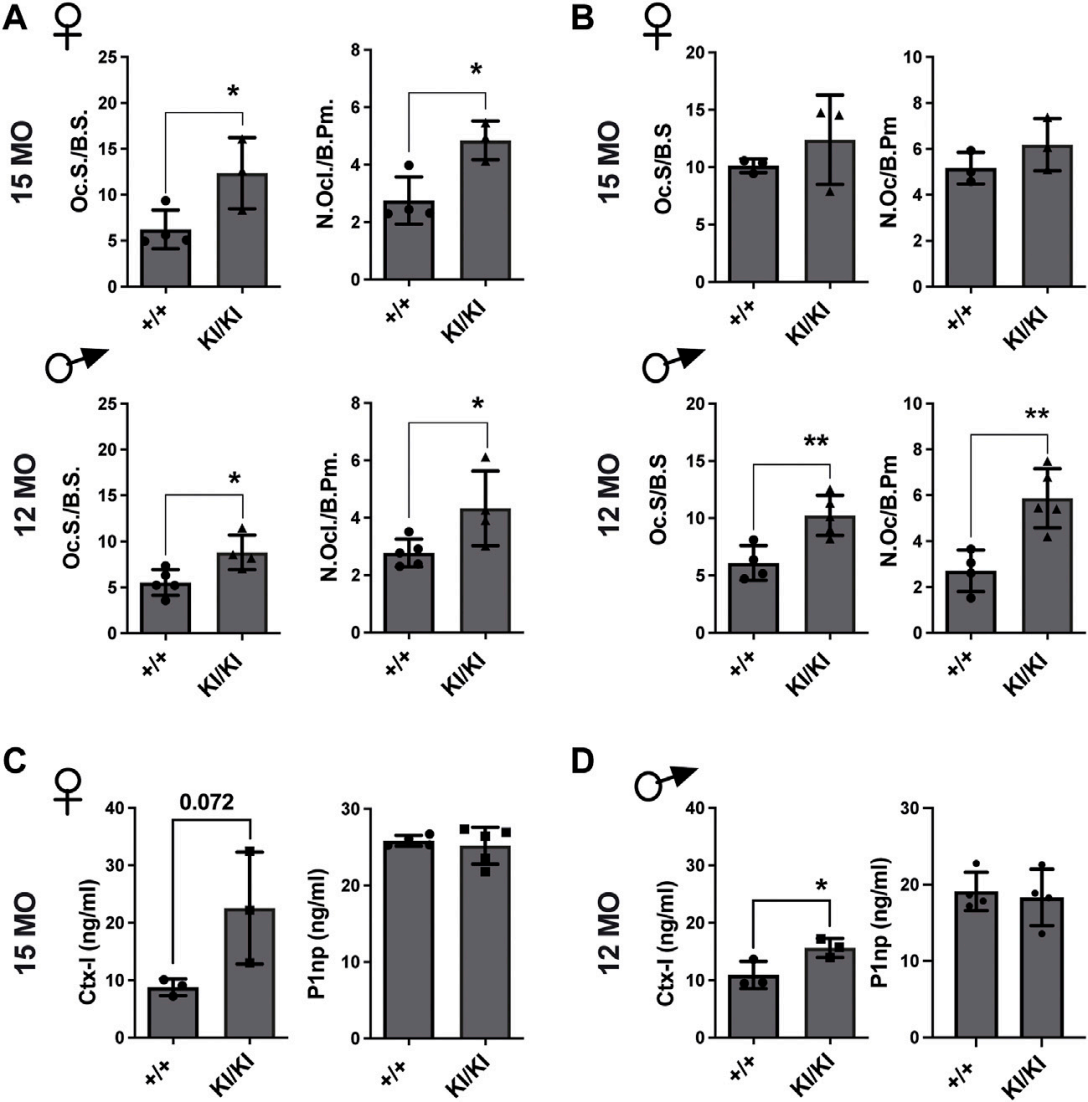
Femoral bone loss in CS 12- and 15-month-old mice is an osteoclast-driven pathology **(A)** TRAP analyses of femoral cortical bone collected from CS 12-month-old males and 15-month-old females. **(B)** TRAP analyses of femoral trabecular bone in CS 12-month-old males and 15-month-old females **(C)** Bone formation (P1NP) and bone degradation (CTX-I) products analyses in 15-month-old-females and **(D)** 12-month-old-males, respectively. 15-month-old-females (*n* = 3–4) and 12-month-old-males (*n* = 4–5). Data are shown as mean ± SEM. Statistical differences were analysed by unpaired 2-tailed student’s t-test where *p < 0.05, **p < 0.01.

Next, we investigated the number of osteoclasts, osteoblasts and osteocytes in femur sections collected from the aged CS KI/KI female and male mouse cohorts. TRAP staining revealed that in contrast to the wild type control mice, osteoclasts surface and number were increased in 15-month-old females and 12-month-old KI/KI males in both femoral cortical and trabecular bone (Figures 4A,B and Supplementary Figure S3).

Osteoblasts and osteocyte numbers were not different within the femoral cortical bone (Supplementary Figure S4). However, we saw a reduced osteoblast number in the trabecular bone of 15-month-old KI/KI females compared to wild type controls, while 12-month-old males showed no difference in osteoblast parameters (Supplementary Figure S4).

Subsequently, we tested whether the CS osteoclasts are functional and are enhancing bone resorption. We observed increased levels of bone degradation product C-terminal telopeptide of type I collagen (Ctx-I) in KI/KI mice, indicating enhanced resorption and thus corroborating with the quantification of osteoclast numbers in same mice. In contrast, bone formation product procollagen type I N-propeptide (P1np) levels in plasma were not changed (Figures 4C,D). These data indicated that an increased osteoclastogenesis underlies bone loss in CS *Hras G12V* mice.

### *CS Hras G12V* mutation enhances *in vitro* osteoclast differentiation

**FIGURE 5:**
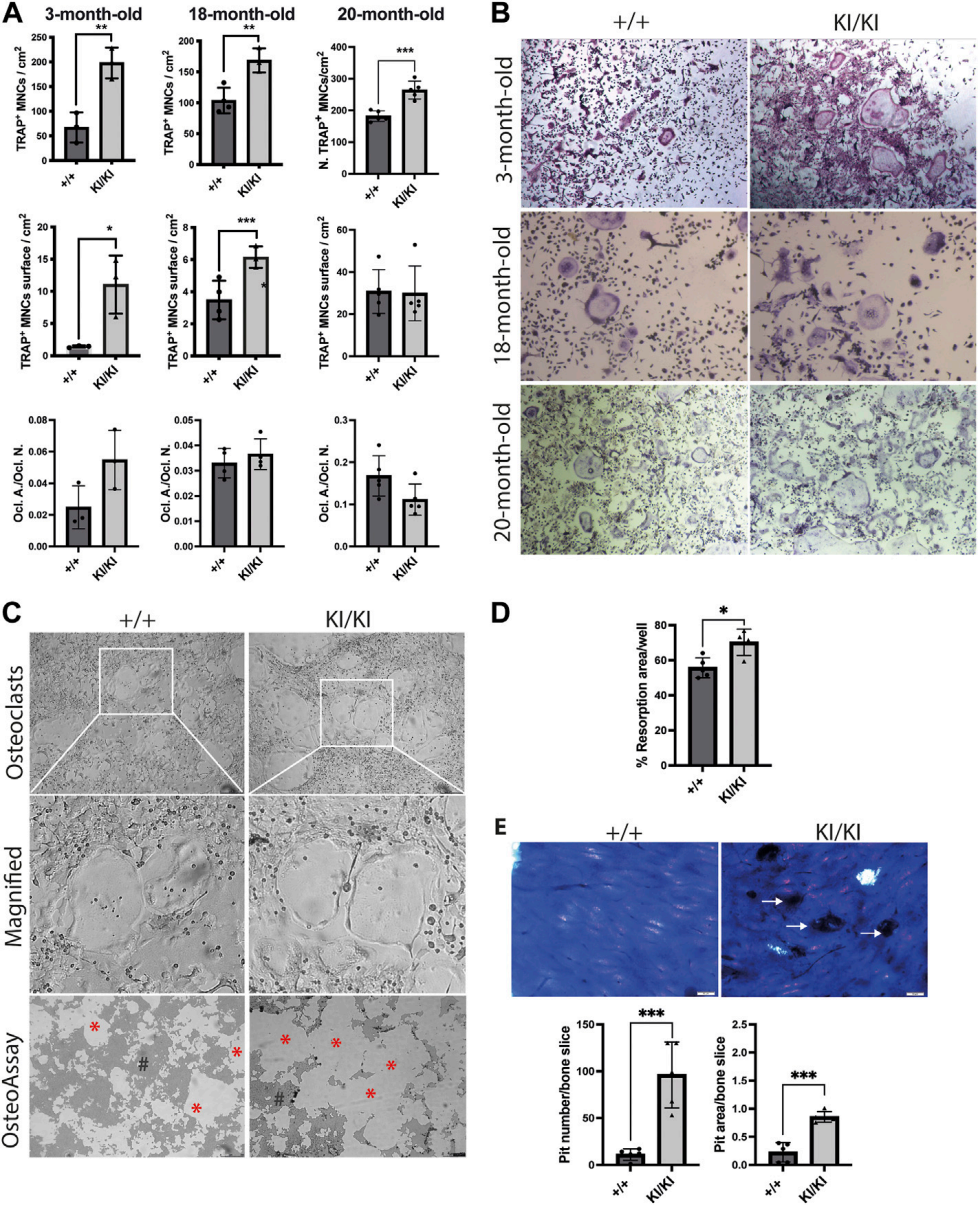
CS *Hras G12V* mutation enhances osteoclast differentiation in a cell autonomous and a donor mouse age-independent mechanism and CS KI/KI osteoclast **(A)** TRAP quantification of osteoclast number, osteoclast surface, the ratio between osteoclast number and osteoclast surface and **(B)** representative images of osteoclasts derived from bone marrow cells collected from various CS age groups. Cells were imaged at ×5 magnification with Leica DMIL LED microscope (Leica) **(C)** Representative images from OsteoAssay resorption assay imaged at ×5 magnification with Leica DMIL LED microscope (Leica) for osteoclasts and with Leica DMI6000B fully automated inverted microscope for resorption areas. Red stars (*) mark the resorbed area on OsteoAssay surface plates while # mark the non-resorbed area. Scale bar 250 µm **(D)** Resorption pit analysis of 15-month-old CS bone marrow cells on Osteo Assay Surface plates. **(E)** Representative images and resorption pit analysis of 15-month-old CS bone marrow cells on bovine cortical bone slices. Representative images of resorption pits on bone slices imaged with Olympus BX41 microscope at ×4 magnification. Scale bar 50 µm. 3-month-old-mice (*n*= 3), 15-month-old-mice (*n* = 4), 18-month-old-mice (*n* = 3–4), 20-month-old-mice (*n* = 5). Data are shown as mean ± SD. Statistical differences were analysed by unpaired 2-tailed student’s t-test where *p < 0.05, **p < 0.01, ***p < 0.001.

Given that osteoclast number was increased in CS mice femur, we next tested the differentiation of osteoclast *in vitro*. To this end, bone marrow cells were collected from 3-, 18- and 20-month-old CS and control mice and were exposed to exogenous M-CSF and RANKL and osteoclast number was monitored by TRAP staining. Quantification of TRAP-positive multinucleated cells indicated that the number of osteoclasts were increased in both CS genotypes when compared to cells isolated from wild type controls, irrespective of donor mouse age (Figures 5A,B). Quantification of total area covered by osteoclasts in a well revealed that osteoclasts derived from 3- and 18-month-old CS mice had an increased area, while the osteoclast-covered area was comparable between osteoclasts derived from 20-month-old CS mice and age matched controls (Figure 5A). Further, the ratios between osteoclast-covered area and osteoclast number revealed no significant differences in the median size between mutant and wild type osteoclasts, irrespective of their age (Figure 5A). These data proves that an increased osteoclastogenesis in CS cells is independent of the donor mouse age and that *Hras G12V* mutation increases osteoclast number without affecting osteoclast size.

In contrast to our findings in osteoclastogenesis, the differentiation into osteoblasts of stromal cells collected from mice of various ages mice was not affected by Hras mutation (Supplementary Figure S5), indicating that Hras G12V *in vitro* effects on bone cells are restricted to osteoclast differentiation, whereas osteoblast differentiation is not affected.

### *Hras G12V* mutation does not affect the resorptive function of KI/KI mutant osteoclasts

Next, we tested osteoclast resorbing activities *in vitro*. To this end, bone marrow cells collected from 15-month-old mice were differentiated into osteoclasts either in plates that either contain bovine cortical bone fragments or on inorganic calcium phosphate substrate. Measurements of resorption area performed on phosphate-coated plates revealed that cells isolated from KI/KI had a significant increase in substrate resorption (Figures 5C,D). Similarly, quantification of resorption pits on bone slices revealed an increased number and area of resorption pits generated by osteoclasts derived from KI/KI mice (Figure 5E).

### MAPK and PI3K inhibitors supress CS osteoclast differentiation *in vitro*

Osteoclast differentiation is a tightly regulated process and two of major RAS-controlled pathways, MAPK and PI3K respectively, are implicated in this process. As osteoclastogenesis is increased in CS, we next tested whether inhibition of both pathways affects the differentiation of KI/KI osteoclasts. Prior to monitoring CS osteoclastogenesis, pilot experiments using wild type bone marrow cells indicated that when we treated bone marrow cells at the same time with MEK inhibitor (MEKi) and the differentiation cocktail, osteoclast differentiation is abolished (Supplementary Figure S6A). On the other hand, the addition of MEK inhibition at preosteoclast stage, reduced osteoclast differentiation without completely blocking differentiation (Supplementary Figure S6B) and was taken as a preferred time point for studying the effect of both types of inhibitors on reversing CS mutation induced osteoclast phenotype. Also, in addition to MEKi, we also tested PI3K inhibitor (PI3Ki) and we observed similar inhibitory effects on osteoclast differentiation (Supplementary Figure S7).

**FIGURE 6:**
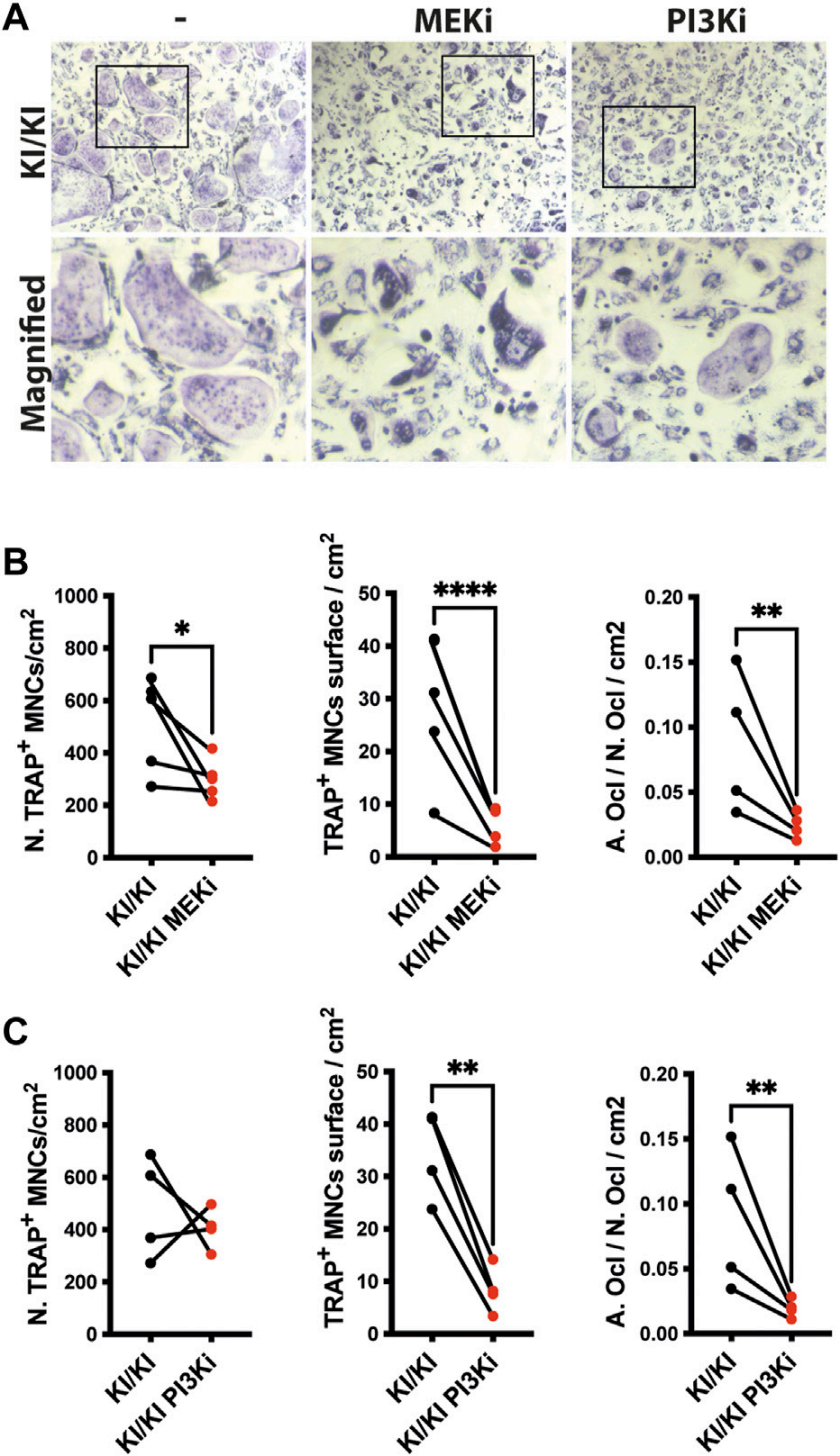
MEK and PI3K inhibitors inhibit in vitro osteoclastogenesis of CS mutant bone marrow cells. **(A)** Representative images of TRAP-stained osteoclasts differentiated in the absence and presence of inhibitors. Images were acquired using Leica DMI6000B microscope at 5X magnification. TRAP based quantification of CS osteoclast number and surface in bone marrow cells isolated from 15-month-old CS mice (n =4) treated with **(B)** MEK inhibitor and **(C)** PI3K inhibitor. Statistical differences were analysed by paired one-tailed student’s t-test where *p < 0.05, **p < 0.01, ***p < 0.001.

Triggered by this observation, we tested the effects of inhibitors on bone marrow cells collected from 15-month-old mutant mice and assessed their differentiation into osteoclasts. Treatment in KI/KI mutant cells revealed that MEK inhibition reduced osteoclast number and size, while PI3K inhibition had inhibitory effect only on osteoclast size (Figures 6A–C).

## Discussion

While previous literature mainly focused on CS as a developmental disorder and its health impact due to congenital abnormalities or disturbed development during childhood, it is increasingly becoming evident that this disorder affects patients’ health throughout their life. Of particular concern is that adult patients develop premature aging-like features, and osteoporosis is a frequent pathology observed in CS. Here, we attempted to study the cellular basis of osteoporosis in CS using the *Hras G12V* mouse model (Schuhmacher et al., 2008). In hetero- and homozygous mutant mice, we observed bone loss in certain age groups with generally a pronounced phenotype with increased *Hras G12V* gene dosage. Our unpublished data indicate that Hras activation in response to G12V germline mutation reduces lifespan in CS mouse in a gene-dosage dependent manner (Chennappan S et al, unpublished data). In addition to a gene dosage-dependent decrease of lifespan, *Hras G12V* mouse model showed a significant bone loss associated with osteopenia/osteoporosis and our study is the first long-term study on bone phenotype using same or other CS mouse models (Schuhmacher et al., 2008; Chen et al., 2009; Schreiber et al., 2017; Oba et al., 2018). Bone histomorphometry and *in vitro* differentiation of bone marrow cells into osteoclasts revealed an increased osteoclastogenesis due to the *Hras G12V* mutation, indicating deregulated osteoclastogenesis as a major contributor to the bone loss in CS*.* Further, *in vitro* treatment with either MAPK or PI3K inhibitors reduced osteoclast differentiation thus proving that activation of Hras G12V and the subsequent activation of both major signalling axes is critical for osteoclast differentiation.

### Gene dosage-dependent effects on bone homeostasis

An intriguing aspect from our CS *Hras G12V* mouse studies is that out of both mutant genotypes an increased gene dosage as in the case of KI/KI has more dramatic effects, not only related to bone loss, but to the overall pathophenotype strength. CS KI/KI mice have the strongest reduction of the lifespan and they were affected earlier in life by numerous pathologies when compared to their +/KI counterparts. Also, CS KI/KI mice have a reduced body weight when compared to wildtype and +/KI counterparts already before weaning (Chennappan S. *et al*, unpublished data). Since bone mass is major contributor to body weight, future analyses in prepubertal and adolescent mice will dissect whether bone mass is reduced early in life and what are the cellular bases underlying a reduced bone mass. CS patients are heterozygous for HRAS mutation, but throughout our studies KI/KI consistently recapitulate much better patients’ pathologies. In contrast to CS patients that are heterozygous for HRAS mutation and display osteoporosis, in mice, bone loss occurred significantly only in CS KI/KI mice, while in +/KI mice these effects were mild at best because a single mutated allele may not have the strength to induce visible pathologies in mice (Figures 2, 3; Supplementary Figures S1, S2). This strong KI/KI pathophenotype strength was also previously reported in the same mouse model where CS brain pathologies were clearly phenocopied in KI/KI mice (Schuhmacher et al., 2008; Viosca et al., 2009; Schreiber et al., 2017). Homozygous mice harbouring Noonan syndrome mutations for either *Kras V14I* (Hernández-Porras et al., 2014) or *Sos1 E846K* (Chen et al., 2010) mutation display pathological features similar to Noonan syndrome patients generally in homozygous background and pathologies strength develops in a gene dosage-dependent manner.

Currently, we do not have a clear explanation about this phenomenon, but we can speculate that *Hras* mutation in a heterozygous background may not be potent enough to enhance osteoclastogenesis and trigger bone loss in +/KI mice.

### Is *Hras G12V* mutation-induced senescence affecting bone cells homeostasis?

Our data *in vivo* and *in vitro* indicated that an increased osteoclastogenesis is a major contributor to the bone loss in CS mice. *In vitro* analyses revealed that this mechanism is cell autonomous when RANKL is provided exogenously, but *in vivo* data is not as consistent across studied groups. Interestingly, aging studies revealed that senescence occurs in osteoblasts and osteocytes and elimination of senescent cells by senolytic drugs reduces osteoclastogenesis and improves bone mass (Farr et al., 2016; Kim et al., 2020). Considering that Hras G12V triggers oncogene-induced senescence in various cell types (Yaswen and Campisi, 2007; Santoriello et al., 2009; Engler et al., 2021), we cannot exclude that *Hras G12V* mutation could trigger senescence in osteoblasts and osteocytes. Therefore, in CS mice that develop osteoporosis and displayed an increased number of osteoclasts, a possible co-occurrence of senescence in bone cells cannot be excluded. Senescent cells are characterised by a senescence-associated secretory phenotype (SASP), whose components such as IL-6, that may stimulate osteoclast differentiation (Farr et al., 2016; Farr et al., 2017).

Another intriguing aspect revealed by bone histomorphometry is that generally bone mineral densities and osteoblast and osteocyte numbers are not affected in CS mouse bones. Nevertheless, we cannot exclude that CS *Hras* mutations do not affect their proper function, either by inducing senescence or through other unknown pathomechanisms. Hras is known to affect collagen, extracellular matrix deposition and elastin (Yamagata et al., 1996; Hinek et al., 2000; Choi et al., 2021), and thus influence bone strength leading to frailty and increased risk for fractures. Therefore, to confirm that Hras does not affect bone strength in mice where bone loss is absent, a functional assay such as 3-point bending test (Deckard et al., 2017; Prodinger et al., 2018) would provide valuable information (Jameson and De Groot, 2010; Biver, 2021).

### MAPK and PI3K inhibition as approaches to rescue bone phenotype in CS mouse

*Hras G12V* mutations lead to its constitutive activation and subsequently to an enhanced activation of the MAPK and PI3K pathways (Castellano and Downward, 2011). Previous studies of these pathways established their role on osteoclast differentiation by enhancing proliferation of monocytic progenitor cells or by controlling subsequent steps such as pre-osteoclasts fusion (Miyazaki et al., 2000; Lee et al., 2009; Heeseog et al., 2010; Moon et al., 2012; Pennanen et al., 2021).

Our attempt to inhibit osteoclastogenesis *in vitro* in CS *Hras G12V* mutant cells established that Hras is a critical regulator of osteoclastogenesis by modulating both MAPK and PI3K pathways, respectively. MEK inhibitor treatment either in progenitor cells or preosteoclasts indicated that Hras-MAPK pathway controls osteoclast differentiation throughout different stages, clearly indicating that dysregulated Hras-MAPK signalling affects bone homeostasis by controlling osteoclast differentiation and MEK inhibitors are feasible as phenotype rescue in future CS mouse studies. Clinically approved MEK and PI3K inhibitors were already used in another CS *Hras G12V* mouse model with only the MEK inhibitor effectively rescuing the skeletal myopathy (Tidyman et al., 2021). Because our *in vitro* data shows that both inhibitors reduced osteoclast differentiation, future studies using both inhibitors in the *Hras G12V* mouse model will be performed to identify whether both pathways can be targeted alone or in combination to preventing bone loss in the CS.

Our study revealed that Hras GTPase is implicated in the regulation of osteoclastogenesis. Due to its constitutive activation in the CS, Hras G12V triggered bone loss by enhancing osteoclastogenesis in the CS *Hras G12V* mouse model. Our data indicated that *in vitro* osteoclastogenesis is increased irrespective to the donor CS mouse age suggest, thus suggesting that *in vivo* osteoclastogenesis requires exogenous factors that may be provided either by bone cells niche or systemically produced in response to Hras G12V constitutive activation. Our experiments using inhibitors of MAPK and PI3K pathways revealed that pathological osteoclastogenesis can be reversed *in vitro* by inhibition of either of these pathways and it may provide a starting point for future phenotype rescue attempts in the CS *Hras G12V* mouse model.

## Data Availability

The original contributions presented in the study are included in the article/Supplementary Material, further inquiries can be directed to the corresponding authors.
