# Supplementary material for: Increased osteoclastogenesis contributes to bone loss in the Costello syndrome Hras G12V mouse model
Source: Front Cell Dev Biol. 2022 Oct 18;10:1000575. doi: 10.3389/fcell.2022.1000575 (PMC9624175; doi:10.3389/fcell.2022.1000575)
Supplement: Supplementary file 1 [file DataSheet1.pdf]

Supplementary Material

1 Supplementary Data

Supplementary methods

Osteoblast *in vitro* differentiation and alkaline phosphatase (ALP) staining

Bone marrow-derived cells containing stromal cells were isolated from mice femur and tibia. 500 000 cells/well were seeded in 24-well plate and were grown to 80-90 % confluency. To induce osteoblast differentiation, α-MEM media containing 1 % penicillin/streptomycin was supplemented with an osteogenic differentiation cocktail containing ß-Glycerophosphate (5 mM) and ascorbic acid (100 µg/ml). Fresh differentiation media was added changed every third day until osteoblasts were harvested after 10 days for qualitative and quantitative ALP staining.

Differentiated osteoblasts were washed 3 times with 1X PBS, fixed with 4 % PFA for 15 minutes, followed by a qualitative ALP staining using ALP staining kit (Sigma Aldrich) according to manufacturer's instructions. Images of plate and individual wells were aquired using Canon EOS 600D camera. Cell viability was determined by Presto Blue Assay (Life Technologies) and the absorbance was measured at 570 nm and the reference wavelength was 595 nm by Nanodrop 2000 (Fisher Scientific GmbH). Quantitative ALP staining was done using AmpliteTMColorimetric ALP assay kit following the manufacturer's instructions and the absorbance was measured at 405 nm by Nanodrop 2000 (Fisher Scientific GmbH).

2 Supplementary Figures

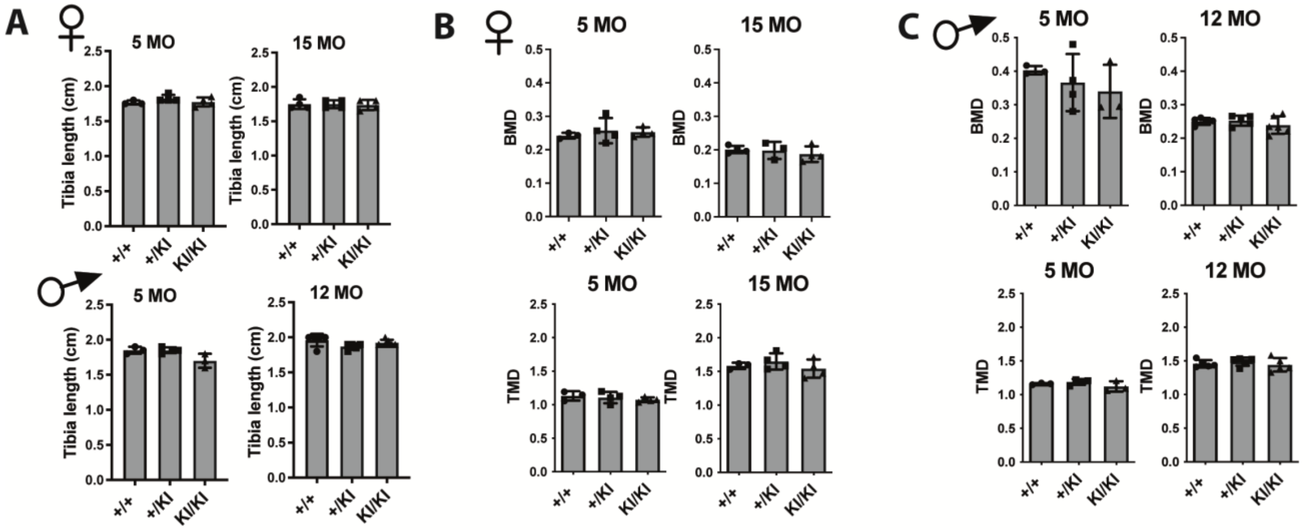

Supplementary figure S1. CS mice show no differences in tibia length and BMD (bone mineral density) and TMD (tissue mineral density) determined by µCT. (A) Tibia length in CS mice. (B) TMD in CS males and females. (C) BMD in CS males and females. 5-month-old-females(n=3-4), 15-

month-old-females (n=4), 5-month-old-males (n=3-4), 12-month-old-males (n=4-5). Data are shown as mean ± SEM.

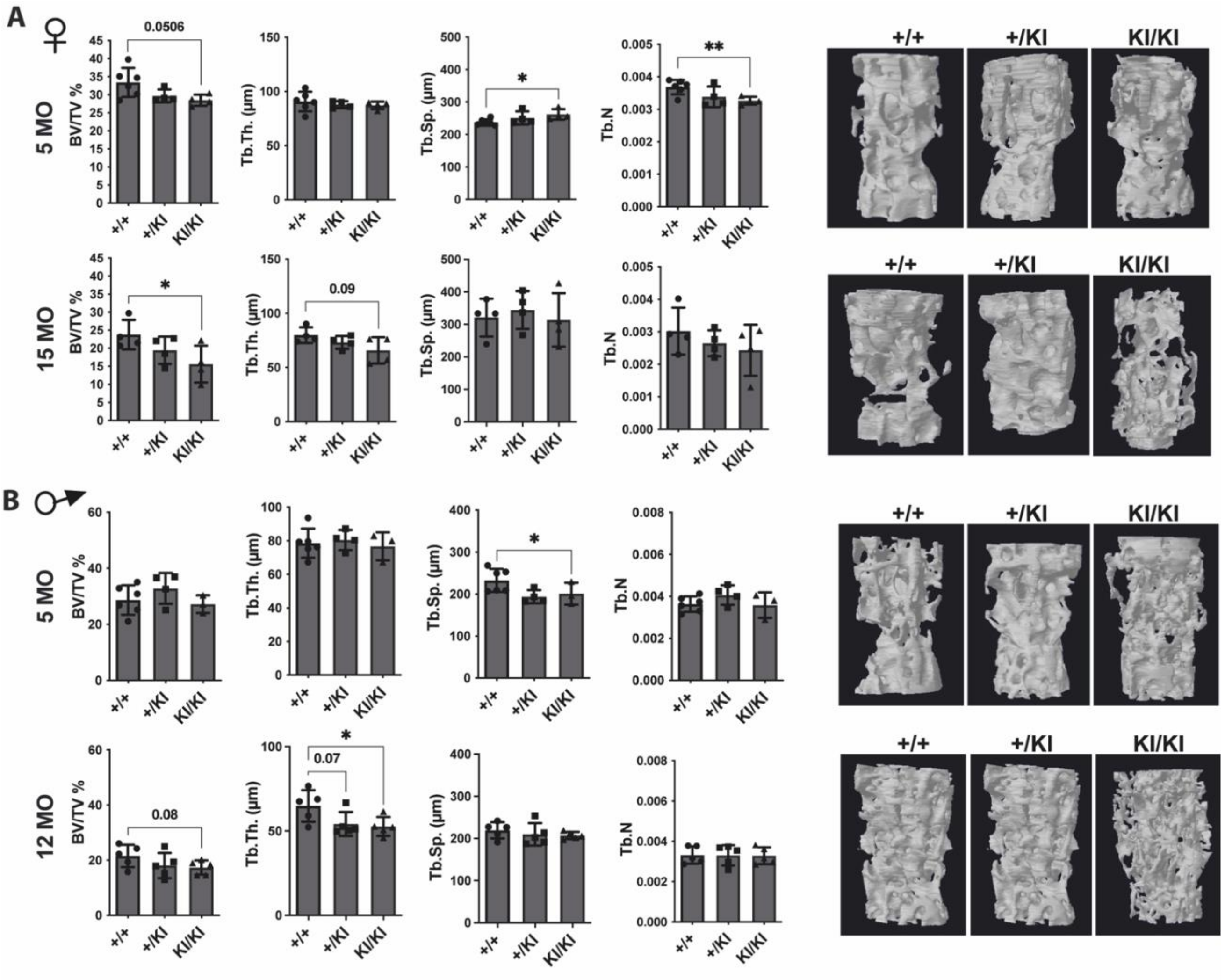

Supplementary figure S2. CS mouse caudal C1 vertebrae are affected by bone loss determined by μCT. Cancellous bone parameters and three-dimensional reconstruction of C1 vertebra of (A) female and (B) male CS mice: 5-month-old-females (n=3-4), 15-month-old-females (n=4), 5-month-old-males (n=3-4), 12-month-old-males (n=4-5). Data are shown as mean ± SEM. Statistical differences were analyzed by unpaired 2-tailed student's t-test where *P<0.05.

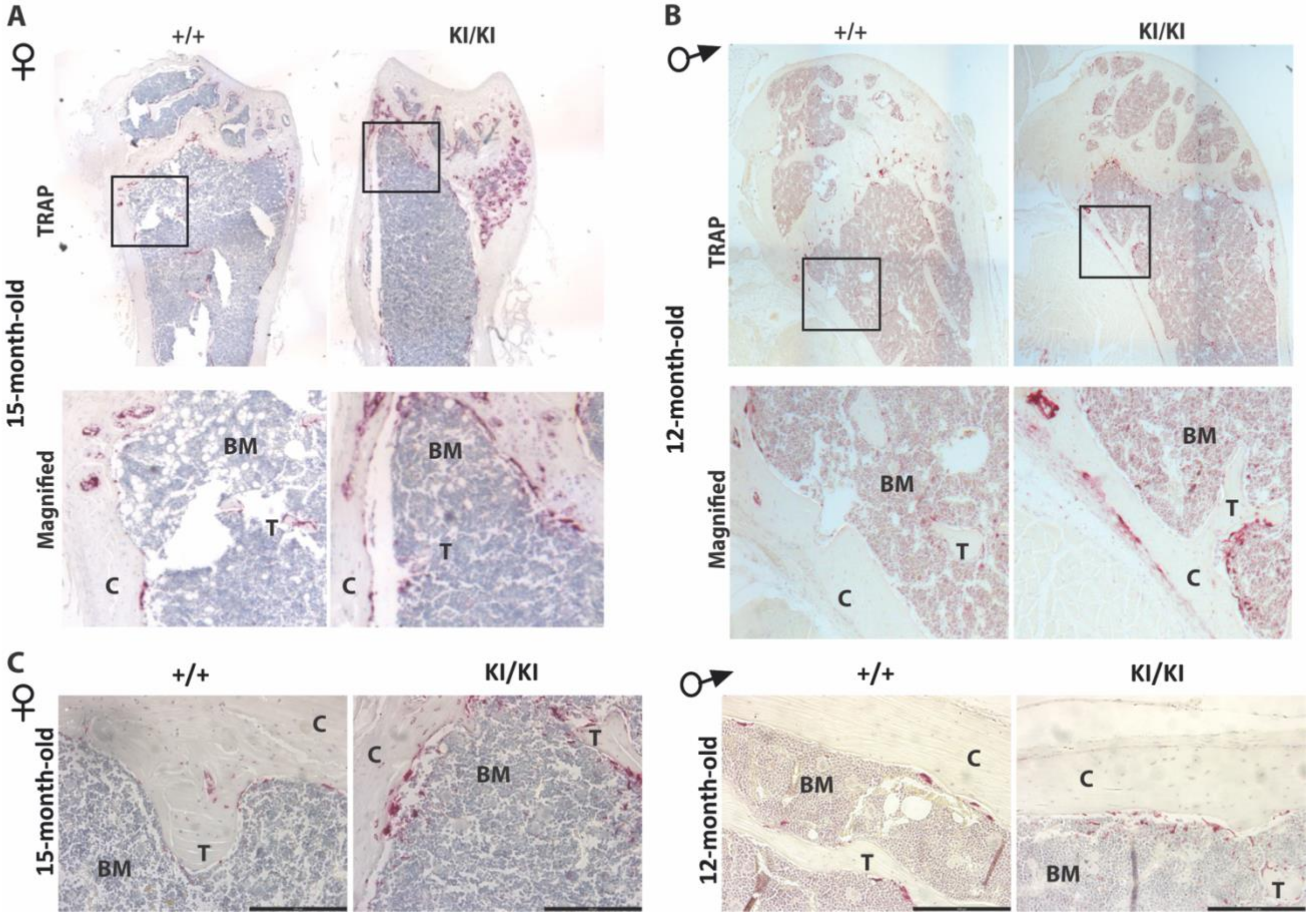

Supplementary figure S3. TRAP staining of the femoral bone. Representative images of TRAP-stained femurs collected from 15-month-old female and 12-month-old male mice. Sections were imaged at (A) 5x magnification and (B) 20x magnification with Leica DMIL LED microscope (Leica). Scale bar (B) 200 µm. Abbreviations: C, cortical femur; T, trabecular femur; BM, bone marrow.

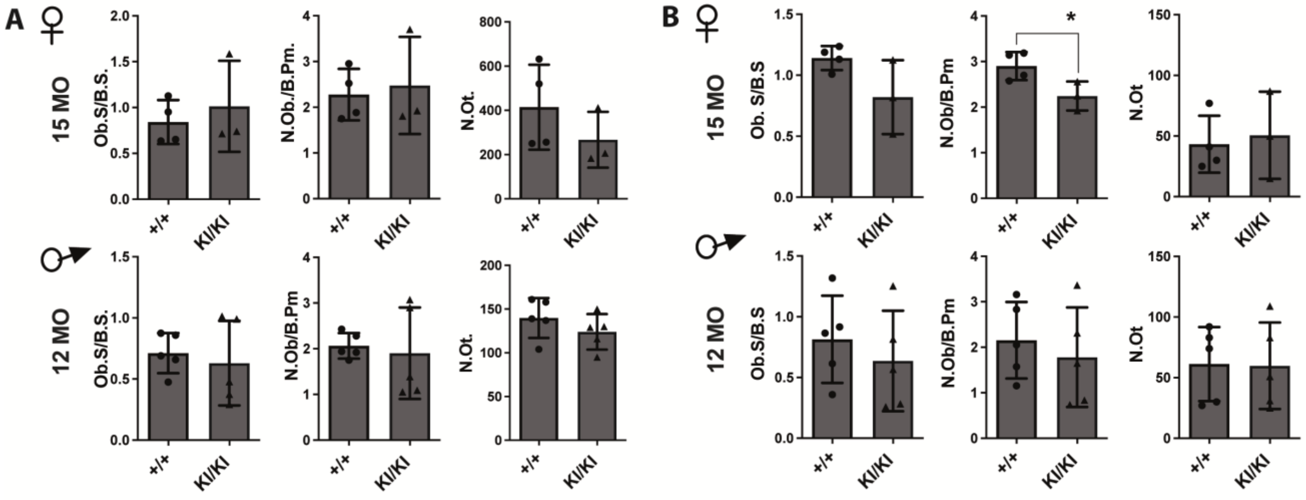

Supplementary Figure S4. Histomorphometry of osteoblasts and osteocytes in CS femoral (A) cortical and (B) trabecular bone. 15-month-old-females (n=3-4) and 12-month-old-males (n=5). Data are shown as mean ± SD. Statistical differences were analyzed by unpaired 2-tailed student's t-test where *P<0.05.

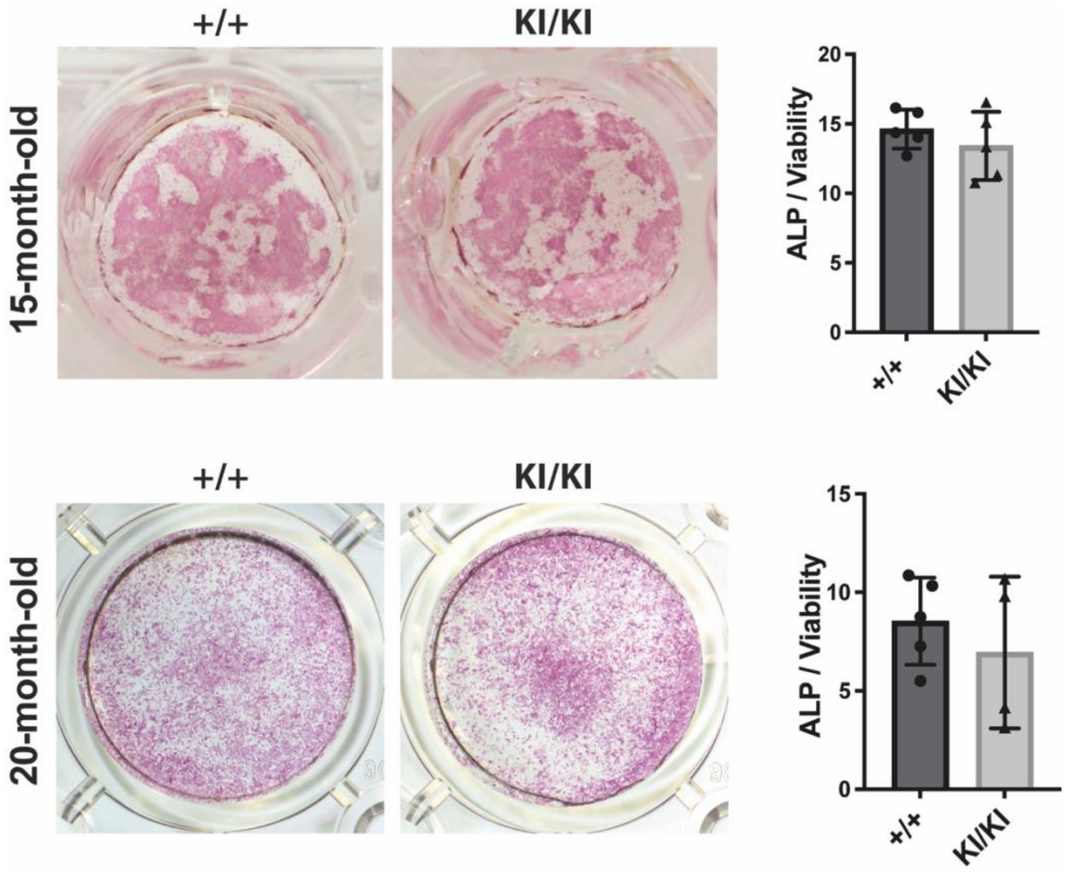

Supplementary figure S5. Bone marrow stromal cells differentiation into osteoblast is not affected by *HRAS G12V* mutations. Qualitative and quantitative ALP staining in 15-month-old CS mice (n=5) and in 20-month-old CS mice and controls (n=4-5). Qualitative ALP staining images of 6-well plates were taken with Canon EOS 600D digital camera. Statistical analyses of the quantification of ALP staining were performed using GraphPad Prism 7 and 8. Data are shown as mean ± SD.

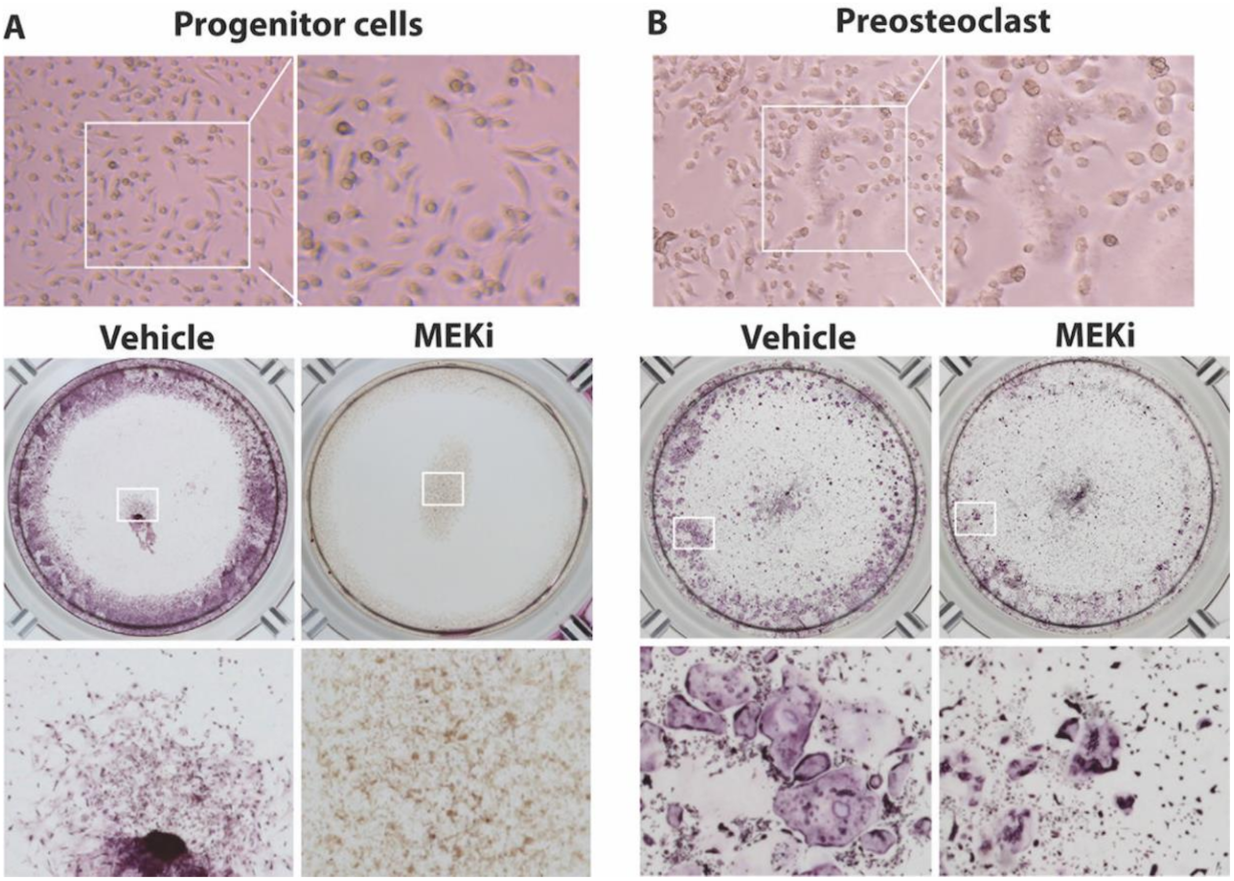

Supplementary figure S6. MAPK inhibition in wild type cells at the stage of (A) progenitor cells and (B) preosteoclasts. TRAP-stained osteoclasts images were taken with Leica DMI6000B microscope, 5X magnification, and TRAP-stained whole wells taken with Canon EOS 600D.

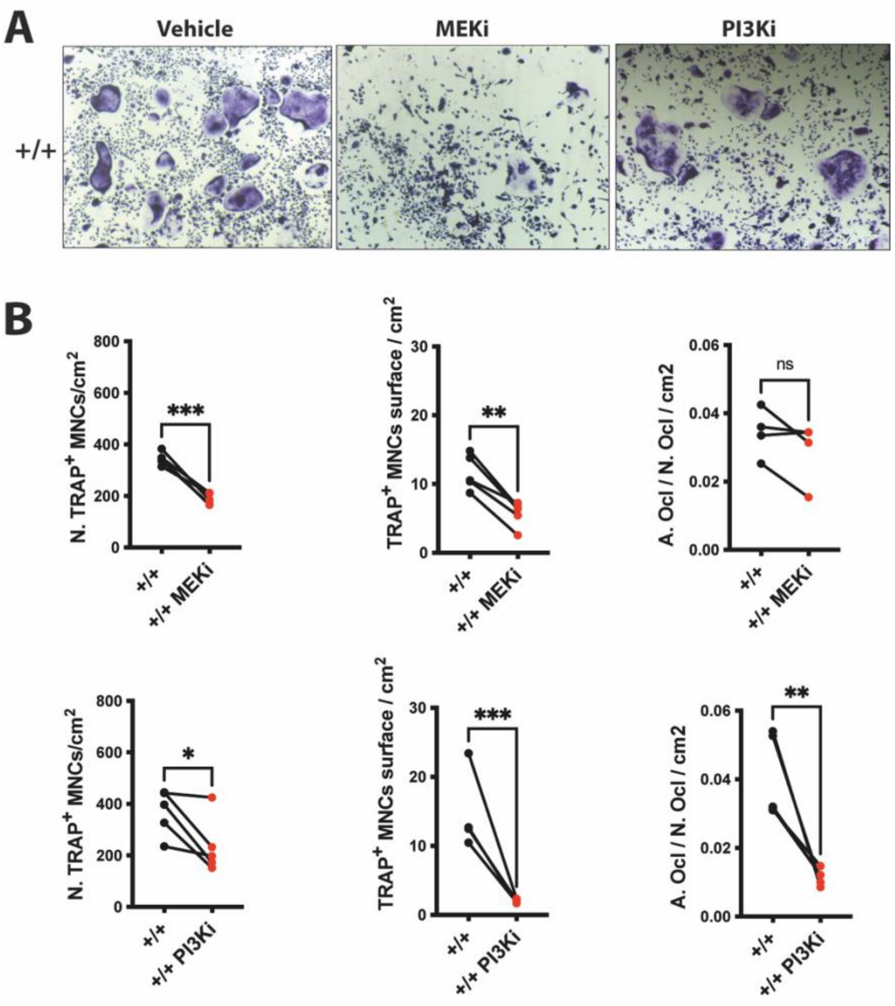

Supplementary figure S7. MEK and PI3K inhibitions reduce osteoclast formation. (A) Representative images of osteoclasts after MEK and PI3K inhibition tests of bone marrow cells collected from wild type mice (+/+) and differentiated into osteoclasts. Images were aquired with Leica DMI6000B with 5X magnification. (B) Statistical analysis of the impact of MEKi and PI3Ki on the differentiation wild type osteoclasts. Data are shown as mean ± SD. Statistical differences were analyzed by unpaired one-tailed student's t-test where *P<0.05, **P<0.01, ***P<0.001.
